# Interleukin 17D Enhances the Developmental Competence of Cloned Pig Embryos by Inhibiting Apoptosis and Promoting Embryonic Genome Activation

**DOI:** 10.3390/ani11113062

**Published:** 2021-10-26

**Authors:** Xiao Wu, Huaxing Zhao, Junkun Lai, Ning Zhang, Junsong Shi, Rong Zhou, Qiaoyun Su, Enqin Zheng, Zheng Xu, Sixiu Huang, Linjun Hong, Ting Gu, Jie Yang, Huaqiang Yang, Gengyuan Cai, Zhenfang Wu, Zicong Li

**Affiliations:** 1 National Engineering Research Center for Breeding Swine Industry, South China Agricultural University, Guangzhou 510642, China; wuxiao5901@stu.scau.edu.cn (X.W.); zhaohuaxing2020@163.com (H.Z.); junkun_lai@163.com (J.L.); zn20182024018@163.com (N.Z.); eqzheng@scau.edu.cn (E.Z.); stonezen@scau.edu.cn (Z.X.); sxhuang815@scau.edu.cn (S.H.); linjun.hong@scau.edu.cn (L.H.); tinggu@scau.edu.cn (T.G.); jieyang2012@hotmail.com (J.Y.); yangh@scau.edu.cn (H.Y.); cgy0415@163.com (G.C.); 2 Department of Animal Genetics, Breeding and Reproduction, College of Animal Science, Guangzhou 510642, China; 3 Guangdong Provincial Key Laboratory of Agro-Animal Genomics and Molecular Breeding, South China Agricultural University, Guangzhou 510642, China; 4 Guangdong Wens Pig Breeding Technology Co., Ltd., Yunfu 527499, China; junsongstone@126.com (J.S.); zr326068732@163.com (R.Z.); 13437867576@163.com (Q.S.); 5 State Key Laboratory for Conservation and Utilization of Subtropical Agro-Bioresources, South China Agricultural University, Guangzhou 510642, China

**Keywords:** SCNT, porcine, IL17D, apoptosis, EGA

## Abstract

**Simple Summary:**

The cloning technique is important for animal husbandry and biomedicine because it can be used to clone superior breeding livestock and produce multipurpose genetically modified animals. However, the success rate of cloning currently is very low due to the low developmental efficiency of cloned embryos, which limits the application of cloning. The low developmental competence is related to the excessive cell death in cloned embryos. Interleukin 17D (IL17D) is required for the normal development of mouse embryos by inhibiting cell death. This study aimed to investigate whether IL17D can improve cloned pig embryo development by inhibiting cell death. Addition of IL17D protein to culture medium decreased the cell death level and improved the developmental ability of cloned pig embryos. IL17D treatment enhanced cloned pig embryo development by regulating cell death-associated gene pathways and promoting genome-wide gene expression, which is probably via up-regulating the expression of a gene called GADD45B. This study provided a new approach to improve the pig cloning efficiency by adding IL17D protein to the culture medium of cloned pig embryos.

**Abstract:**

Cloned animals generated by the somatic cell nuclear transfer (SCNT) approach are valuable for the farm animal industry and biomedical science. Nevertheless, the extremely low developmental efficiency of cloned embryos hinders the application of SCNT. Low developmental competence is related to the higher apoptosis level in cloned embryos than in fertilization-derived counterparts. Interleukin 17D (IL17D) expression is up-regulated during early mouse embryo development and is required for normal development of mouse embryos by inhibiting apoptosis. This study aimed to investigate whether IL17D plays roles in regulating pig SCNT embryo development. Supplementation of IL17D to culture medium improved the developmental competence and decreased the cell apoptosis level in cloned porcine embryos. The transcriptome data indicated that IL17D activated apoptosis-associated pathways and promoted global gene expression at embryonic genome activation (EGA) stage in treated pig SCNT embryos. Treating pig SCNT embryos with IL17D up-regulated expression of *GADD45B*, which is functional in inhibiting apoptosis and promoting EGA. Overexpression of *GADD45B* enhanced the developmental efficiency of cloned pig embryos. These results suggested that IL17D treatment enhanced the developmental ability of cloned pig embryos by suppressing apoptosis and promoting EGA, which was related to the up-regulation of *GADD45B* expression. This study demonstrated the roles of IL17D in early development of porcine SCNT embryos and provided a new approach to improve the developmental efficiency of cloned porcine embryos.

## 1. Introduction

Somatic cell nuclear transfer (SCNT) technique, also called cloning, is important for animal husbandry, biotechnology, biopharmacy and biomedicine because it can be used to clone superior breeding livestock [1,2] and produce multipurpose genetically modified animals [3,4,5]. However, the developmental efficiency of SCNT embryos is extremely low [6,7,8,9], which limits the practical application of cloning.

So far, a broad spectrum of research has characterized the biological, molecular and epigenetic determinants of mammalian SCNT embryo development. The afore-indicated determinants involve: (1) the origin of nuclear donor cells [10,11,12,13,14], (2) the quality of nuclear recipient oocytes determined by the parameters related to meiotic, epigenomic and cytoplasmic maturity [15,16], (3) the methods used to stimulate the embryo-specific developmental program of enucleated oocytes [17,18,19,20], (4) the incidence of programmed cell apoptosis in in vitro-cultured nuclear donor cells and cloned embryos [21,22,23], (5) the intergenomic communication between nuclear DNA and mitochondrial DNA in cloned embryos [24,25,26,27,28,29], and (6) the capacity of donor cell nuclei to be reprogrammed in cloned embryos [30,31,32,33].

Apoptosis is one of the major reasons that affects cloned embryo development. Many studies have shown that the cell apoptosis level in SCNT embryos is higher than that in fertilization-derived counterparts [34,35,36]. Treatment with anti-oxidative molecules, such as vitamin C [37], vitamin E [38], and melatonin [39,40], improve cloned embryo development by inhibiting apoptosis. Cytokines, such as IGF1 [41] and CSF2 [42,43], also regulate apoptosis-related genes to increase the developmental rate of SCNT embryos. In addition, small RNAs, such as miR-101-2 [44] and miR-449b [45], decrease apoptosis to enhance the developmental efficiency of SCNT embryos.

Interleukin 17D (IL17D) is a cytokine that plays critical roles in mouse early embryo development by regulating apoptosis-associated pathways [46]. In fertilization-produced mouse embryos, *IL17D* expression is naturally up-regulated during 2-cell to 4-cell stage, and the inhibition of *IL17D* transcription impairs embryo development by increasing apoptosis. Meanwhile, supplementation of IL17D rescues embryonic developmental defects caused by the inhibition of *IL17D* expression.

The purpose of this study was to investigate whether IL17D plays roles in regulating pig SCNT embryo development. We demonstrated that the addition of IL17D to the culture medium increased the developmental ability of cloned porcine embryos via modulating apoptosis-relevant pathways and global gene expression.

## 2. Materials and Methods

### 2.1. Ethics Statement

This study was performed in accordance with the “Guidelines with Respect to Caring for Laboratory Animals” issued by the Ministry of Science and Technology of China. The animal experimental protocol was approved by the Institutional Animal Care and Use Committee of South China Agricultural University. All efforts were made to minimize the suffering of animals tested.

### 2.2. Medium and Reagents

All the chemicals used in this study were procured from Sigma–Aldrich Company (MO, USA) unless otherwise mentioned.

### 2.3. In Vitro Oocyte Maturation

Porcine ovaries were collected from a local slaughterhouse and transported to the laboratory in 0.9% (*w*/*v*) NaCl solution supplemented with penicillin-G (100 IU/mL) and streptomycin sulfate (100 mg/L) at 30–35 °C. Follicular fluid containing cumulus–oocyte complexes (COCs) was aspirated from 3–6 mm diameter antral follicles by using an 18-gauge needle and syringe. COCs with at least three layers of compact cumulus cells and a homogenous cytoplasm were selected from follicle fluid and incubated with in vitro maturation medium [47] at 38.5 °C with 5% CO_2_ for 42–44 h. Matured COCs were freed from cumulus cells by repeated pipetting in Dulbecco’s phosphate-buffered saline (DPBS) (Gibco, Grand Island, NY, USA) containing 1 mg/mL hyaluronidase. Oocytes with uniform cytoplasm, round cell morphology, and clearly visible polar body were selected as mature oocytes for subsequent experiments.

### 2.4. In Vitro Fertilization

Porcine semen was purchased from a local animal husbandry company. The sperm density was adjusted to 2 × 107/mL with modified Tris-buffered medium(mTBM) [48] for capacitation. The in vitro mature oocytes were washed with mTBM and transferred to a four-well plate with 40–50 oocytes and 500 µL mTBM per well. The capacitated semen (50 µL) was added to obtain 1 × 106 sperm per well. After incubating at 38 °C for 6 h, the fertilized eggs were washed with DPBS supplemented with 1 mg/mL BSA to remove the sperm on the surface. The eggs were then transferred to PZM-3 medium [49] and cultured in an incubator at 38.5 °C and 5% CO_2_. Cleavage rate, blastocyst rate, and total cell number of blastocysts were calculated at 48 and 144 h.

### 2.5. Somatic Cell Nuclear Transfer

Porcine fetal fibroblasts were cultured in DMEM with 10% of FBS at 38.5 °C and 5% CO_2_. When the cells reached a confluence of 90–100%, they were digested with trypsin and resuspended in in vitro manipulation medium for later use. Mature oocytes were denucleated by blind aspiration. One donor cell was injected through the enucleated incision into the perivitelline space of each enucleated oocyte. Reconstituted embryos were cultured in PZM-3 at 38.5 °C for 4 h and 5% CO_2_. The reconstructed embryos were activated in the fusion solution [1] through two direct current pulses of 150 V/mm for 50 ms. The reconstituted embryos were transferred to PZM-3 and cultured in an incubator at 38.5 °C and 5% CO_2_. The numbers of cleaved embryos and blastocysts were calculated at 48 h and 144 h post-activation, respectively. Blastocysts were defined as embryos that contain a fluid-filled blastocoel and have at least 20 cells.

### 2.6. IL17D Treatment

Human IL17D protein (Cat no. CG96, Novoprotein, Fremont, CA, USA), which has 98% homology to porcine IL17D in amino acid sequences, was added to the PZM-3 culture medium of porcine IVF and SCNT embryos at 1-cell stage (right after fertilization of IVF embryos or activation of SCNT embryos) or 4-cell stage (48 h post fertilization or activation of SCNT embryos). After culturing for 48 h, the medium containing IL17D was replaced by PZM-3 medium. The experimental design and groups of IL17D treatment is shown in Table 1.

### 2.7. Transcriptome Sequencing

Transcriptome sequencing was performed as previously described [50]. Twenty to thirty four-cell stage embryonic cells of the same treatment group were mixed as one sample and collected into a 1 mL tube containing 3 µL of lysis buffer with RNase inhibitor. Complementary DNA (cDNA) was generated and amplified for sequencing based on smartseq2 method. After cDNA amplification and purification, the overall quality of the initial cell was performed with an Agilent 2100 Bioanalyzer. We determined the degradation of the sample according to the RNA integrity number by Agilent Bioanalyzer. The clustered library preparations were then sequenced on an Illumina Nova platform, and 150-bp paired-end reads were generated. Raw read data were the original RNA-Seq reads which used Trimmomatic to filter low-quality reads and trim the linker sequence [51]. Clean read data were then aligned to the susScr11 reference genome using STAR [52]. Reads aligned to genes are counted by cufflinks (v2.2.1). The fragments per kilobase of exon model per million mapped reads (FPKMs) are normalized using cuffnorm. Differentially expressed genes are calculated using cuffdiff [53]. Differentially expressed genes between two groups (*p* < 0.05) were filtered by [log2 FoldCharge] > 1 and FPKM > 5. Raw RNA-Seq data for 2-cell and 4-cell stage IVV Duroc embryos, SCNT Duroc embryos, and SCNT Laiwu embryos, were obtained from the Gene Expression Omnibus (GEO) datasets (accession no. GSE125706) [54].

### 2.8. Quantitative Real-Time Polymerase Chain Reaction (qRT-PCR)

Total RNAs were extracted from 4-cell stage embryos and blastocysts by using the Qiagen AllPrep DNA/RNA Micro Kit (Qiagen, Gaithersburg, MD, USA) according to the manufacturer’s instructions. cDNA was generated by the PrimeScript RT reagent kit with gDNA Eraser (TAKARA, Shiga, Japan). The synthesized cDNA was used for qRT-PCR by PowerUp SYBR Green Master Mix (Thermo Fisher Scientific, CA, USA). qRT-PCR was performed on QuantStudio™ 7 Flex Real-Time PCR System (Thermo Fisher Scientific) by following the parameters recommended by the manufacturer. The reactions were run and performed in triplicate as previously described [55]. GAPDH was used as the house-keeping gene, and the relative mRNA expression was calculated using the 2-ΔΔCt method. The primer sequences are shown in Table 2. The amplification efficiency of each pair of primers at different cDNA template concentrations was validated to be >95% (Figure S1 and Table S1), following a previously reported method [56].

### 2.9. Terminal Deoxynucleotidyl Transferase (TdT)-Mediated 2′-Deoxyuridine-5′-Triphosphate-Digoxigenin Nick-End Labeling (TUNEL) Assay

Blastocysts were analyzed by the TUNEL apoptosis detection kit (YEASEN, SH, China). The embryos were fixed in 4% paraformaldehyde and permeated for 30 min in 0.5% Triton X-100. The embryos were incubated in TdT incubation buffer at 38 °C for 1 h. The nuclei were stained with Hoechst33342 at room temperature for 5 min. The stained embryos were placed on slides, and photos were taken under the microscope.

### 2.10. Plasmid Construction

The coding sequences (CDS) of porcine *GADD45B* gene (GenBank no. XM_005654701.2) was synthesized and inserted between the NheI and HindIII sites at the multiple cloning sites of the pcDNA3.1(+)-EGFP vector to generate the pcDNA3.1-GADD45B-EGFP vector.

### 2.11. Microinjection

Microinjection of the porcine *GADD45B* expression plasmid into cloned pig embryos was performed using a micropipette driven by a Piezo (Eppendorf). Ten picoliter of plasmids or water was injected into the cytoplasm of each embryo after activation of SCNT embryos. After microinjection, the embryos were transferred to PZM-3 and cultured in an incubator at 38.5 °C and 5% CO_2_.

### 2.12. Statistical Analysis

SPSS software version 20 was used for statistical analysis. A Chi-square test was performed to determine differences in cleavage rate, blastocyst rate, and percentage of apoptosis cells. One-way ANOVA was performed to evaluate differences in gene expression level and total cell number of blastocysts.

## 3. Results

### 3.1. IL17D Expression Is Abnormal in Cloned Pig Embryos Compared to That in In Vivo-Fertilization-Derived Pig Embryos

**Figure 1:**
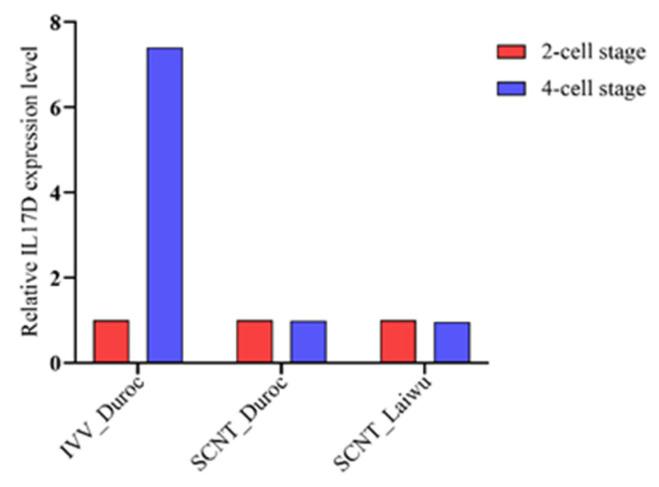
Relative *IL17D* mRNA expression level in 2-cell and 4-cell stage IVV Duroc embryos, SCNT Duroc embryos, and SCNT Laiwu embryos.

Analysis of a published transcriptome sequencing data set showed that the expression of *IL17D* mRNA is up-regulated by approximately eight folds in in vivo fertilization-derived (IVV) porcine embryos while it is unchanged in cloned porcine embryos during the 2-cell to 4-cell stage (Figure 1). This suggests that up-regulation of *IL17D* expression during early stage is required for normal porcine embryo development and the expression of *IL17D* in cloned pig embryos is abnormal.

### 3.2. IL17D Treatment Improved the Developmental Competence of Porcine SCNT and IVF Embryos

The activated cloned embryos at the 1-cell stage were treated with 5, 25, 50 and 100 ng/mL IL17D for 48 h to examine the effects of IL17D on porcine SCNT embryo development. Treatment with 50 ng/mL IL17D significantly enhanced the blastocyst rates of porcine SCNT embryos (Table 3). Treatment with 25 and 100 ng/mL IL17D did not significantly improve but tented to improve the blastocyst rate of porcine SCNT embryos (Table 3). The addition of 50 ng/mL IL17D at 1-cell stage for 48 h also significantly enhanced the blastocyst rate of the treated porcine IVF embryos (Table 4). However, treatment with porcine SCNT embryos with 50 ng/mL IL17D at 4-cell stage for 48 h did not significantly affect the developmental efficiency (Table 5).

### 3.3. IL17D Treatment Inhibited the Apoptosis of Porcine SCNT and IVF Embryos at Blastocyst Stage

**Figure 2:**
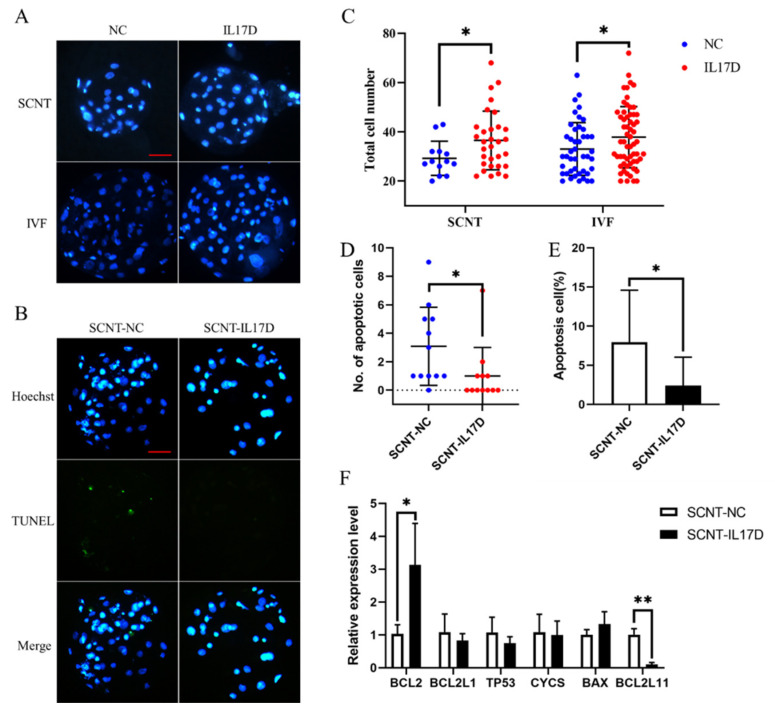
Effect of IL17D treatment on apoptosis of SCNT and IVF embryos. (**A**) Nuclear staining of SCNT-NC, SCNT-IL17D, IVF-NC and IVF-IL17D embryos at blastocyst stage by Hoechst33342 (blue). Bar = 100 µm. (**B**) TUNEL assay of SCNT-NC, SCNT-IL17D embryos at blastocyst stage. Bar = 100 µm. (**C**) Total cell number of SCNT-NC, SCNT-IL17D, IVF-NC and IVF-IL17D embryos at blastocyst stage. (**D**) Number and (**E**) proportion of apoptotic cells of SCNT-NC, SCNT-IL17D embryos at blastocyst stage. (**F**) The mRNA expression level of apoptosis-related genes of SCNT-NC and SCNT-IL17D embryos at blastocyst stage. Data were obtained from three independent replicates. Values were presented as mean ± SEM. * and ** means *p* < 0.05 and *p* < 0.01, respectively.

Treatment with 50 ng/mL IL17D at 1-cell stage for 48 h also significantly increased the total cell number of porcine SCNT and IVF embryos at the blastocyst stage (Figure 2A,C). The number and proportion of apoptotic cells at the blastocyst stage significantly decreased in porcine SCNT embryos treated with IL17D (Figure 2B,D,E). At the blastocyst stage of IL17D-treated porcine SCNT embryos, the mRNA expression of the pro-apoptotic gene *BCL2L11* was down-regulated and the transcription of the anti-apoptotic gene *BCL2* was up-regulated. The mRNA abundance of four other tested apoptosis-related genes was not significantly changed (Figure 2F). These results indicated that IL17D treatment inhibited apoptosis in porcine SCNT embryos.

### 3.4. IL17D Treatment Increased the Expression of Global Genes in Porcine SCNT Embryos at 4-Cell Stage

**Figure 3:**
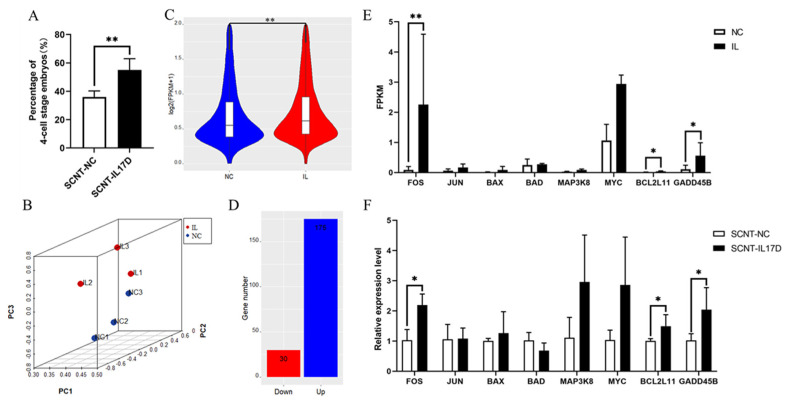
Transcriptome sequencing of SCNT-IL17D (IL) and SCNT-NC (NC) embryos at 4-cell stage. (**A**) Comparison of the percentage of embryos developing to 4-cell stage between SCNT-IL17D and SCNT-NC groups. (**B**) Principal component analysis (PCA) plot for six samples from IL and NC groups at 4-cell stage. Each sample was a mixture of 20 to 30 embryos. (**C**) Violin plot of mRNA levels of 6296 transcriptome sequencing-detected genes (with the sum of individual gene’s FPKM value >1) of IL and NC embryos at 4-cell stage. (**D**) Bar plot of differentially expressed genes (IL vs. NC) between IL and NC groups at 4-cell stage. (**E**) Transcriptome sequencing-detected mRNA expression levels of eight randomly selected genes in NC and IL group. (**F**) qRT-PCR -measured relative mRNA expression levels of the same eight genes in SCNT-NC and SCNT-IL17D embryos at 4-cell stage. Data were obtained from three independent replicates. Values were presented as mean ± SEM. * and ** means *p* < 0.05 and *p* < 0.01, respectively.

We observed that porcine SCNT embryos treated with 50 ng/mL IL17D at 1-cell stage for 48 h exhibited higher developmental rate than the control group at 4-cell stage (Figure 3A). This observation, together with the result shown in Table 5, suggested that the added IL17D in the embryo culture medium exerted or started to exert its effects before the 4-cell stage of porcine SCNT embryos. Therefore, 4-cell stage SCNT-IL17D and SCNT-NC embryos were collected for transcriptome sequencing to compare their differences in gene expression patterns. The PCA results showed that three samples in the SCNT-IL17D (IL) group were separated from the three other samples in the SCNT-NC (NC) group (Figure 3B). This finding implied that the two groups of embryos had different gene expression patterns. The mRNA expression level of transcriptome sequencing-detected genome-wide genes was significantly increased in the IL group compared with that in the NC group (Figure 3C). The analysis of differentially expressed genes (DEGs) showed that in IL embryos, the number of up-regulated genes (175) was higher than the number of down-regulated genes (30) (Figure 3D). Hence, IL17D treatment up-regulated the expression of global genes in porcine SCNT embryos at the 4-cell stage. To confirm the transcriptome sequencing data, we randomly selected eight genes including *FOS*, *JUN*, *BAX*, *BAD*, *MAP3K8*, *MYC*, *BCL2L11*, and *GADD45B* for qRT-PCR verification. The mRNA expression levels of the eight genes examined by transcriptome sequencing matched with those measured by qRT-PCR (Figure 3E,F).

### 3.5. IL17D Treatment Improved Porcine SCNT Embryo Development by Regulating Apoptosis-Related Pathways

**Figure 4:**
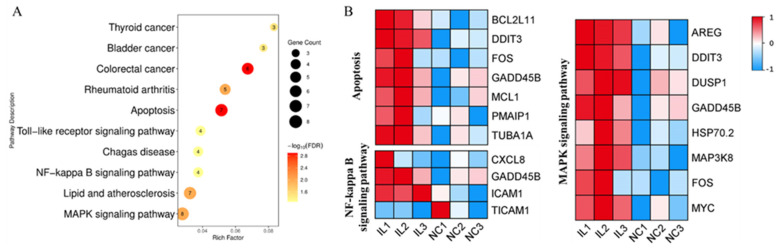
Analysis of transcriptome data of 4-cell stage SCNT-IL17D and SCNT-NC embryos. (**A**) Kyoto Encyclopedia of Genes and Genomes (KEGG) pathway analysis of DEGs between 4-cell stage SCNT-IL17D and SCNT-NC embryos. (**B**) Heatmap of DEGs participated in three apoptosis-related pathways.

Among the top 10 significantly enriched KEGG pathways for DEGs between IL and NC groups, three pathways including apoptosis pathway, MAPK signaling pathway, and NF-kappa B signaling pathway are involved in the IL17 signaling pathway (Figure 4A) [57]. The three IL17-related pathways are also relevant to apoptosis. The transcription levels of most DEGs enriched in the three apoptosis-associated pathways, including pro-apoptotic and anti-apoptotic/pro-survival genes, were up-regulated in IL group compared with those in NC group (Figure 4B) [58]. These results suggested that IL17D signal through the IL17 pathway to act on apoptosis-related genes to enhance cloned porcine embryo development.

### 3.6. The Effects of IL17D on Improving Porcine SCNT Embryo Development Might Be Related to the Up-Regulation of GADD45B Expression

**Figure 5:**
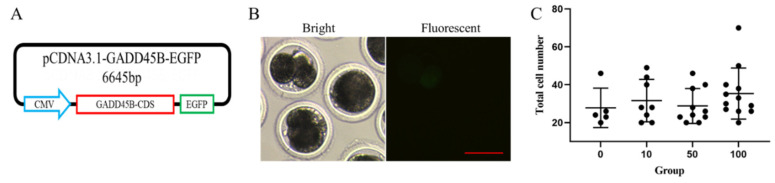
Overexpression of *GADD45B* in cloned pig embryos. (**A**) Map of the porcine *GADD45B* expression plasmid. (**B**) Expression of EGFP marker gene at 2-cell stage of injected cloned pig embryos. Bar = 100 µm. (**C**) Total cell number at blastocyst stage of SCNT embryos injected with *GADD45B* expression plasmid.

The above results suggested that IL17D enhanced pig SCNT embryo development by inhibiting apoptosis and promoting EGA. Among the genes whose expression was up-regulated at 4-cell stage in IL17D-treated pig SCNT embryos, *GADD45B* was noticeable because it not only has anti-apoptotic functions [59,60], but also plays important roles in EGA [61]. To examine whether the effects of IL17D on enhancing pig SCNT embryo development is related to the upregulation of *GADD45B* expression, we constructed a porcine *GADD45B* expression plasmid (Figure 5A) and injected it into cloned pig embryos. Expression of the EGFP marker gene that was linked to the porcine *GADD45B* gene was observed at 2-cell stage of injected pig SCNT embryos (Figure 5B). This suggested that *GADD45B* was overexpressed in injected pig SCNT embryos. Injection of *GADD45B* expression plasmid showed no effect on the total cell number at blastocyst stage of cloned pig embryos (Figure 5C). However, injection of 100 ng/µL of *GADD45B* expression plasmid increased the blastocyst rate of cloned pig embryos and injection of 50 ng/µL and 10 ng/µL of *GADD45B* expression plasmid tended to improve the blastocyst rate of cloned pig embryos (Table 6). This suggested that IL17D improved porcine SCNT embryo development probably by up-regulating *GADD45B* expression.

## 4. Discussion

IL17D enhanced porcine SCNT embryo development by inhibiting apoptosis. The functions of IL17D observed in porcine SCNT embryos are consistent with those reported in mouse IVF embryos [46]. Our transcriptome data showed that the expression levels of pro-apoptotic DEGs such as *BCL2L11* [62], *PMAIP1* [63], and *DDIT3* [64] were up-regulated with anti-apoptotic DEGs such as *GADD45B* [60], *MCL1* [65], and *DUSP1* [66], in SCNT-IL17D embryos compared with those in SCNT-NC embryos. However, the ratio of the expression level of pro-apoptotic genes to that of anti-apoptotic genes might be lower in SCNT-IL17D embryos than in SCNT-NC embryos. This phenomenon might result in the overall anti-apoptotic effect in IL17D-treated SCNT embryos, which is supported by the data of this study.

In early mouse embryos, transcription of *IL17D* is positively associated with the expression of its promoter-associated noncoding RNA (pancil17d), which is oppositely transcribed from the bidirectional *IL17D* promoter and is indispensable for EGA of mouse embryos [46]. This suggested that *IL17D* is involved in EGA. SCNT embryos are associated with defects of embryonic genome activation (EGA) [54,67]. IL17D also promoted EGA in treated porcine SCNT embryos because it increased the expression of global genes at 4-cell stage, which is the major EGA stage for porcine embryos [68,69]. The promotion of EGA in porcine SCNT embryos by *IL17D* might be related to the up-regulation of the expression of *GADD45B*, which is functional in inhibiting apoptosis and promoting EGA [59,60,61]. More importantly, overexpression of *GADD45B* enhanced the developmental ability of cloned pig embryos.

*IL17D* transcription was naturally increased by about eight folds in porcine IVV embryos during 2-cell to 4-cell stage, suggesting that the up-regulation of IL17D before the 4-cell stage is required for normal porcine embryo development. This finding is consistent with our observation that the developmental ability of porcine SCNT embryos was improved by supplementation of IL17D at 1-cell stage but not at 4-cell stage. Nevertheless, the addition of IL17D at the 4-cell stage of mouse IVF embryos rescued the developmental failure induced by the suppression of IL17D expression. The difference in the action time point of IL17D in porcine SCNT embryos and mouse IVF embryos might be related to their differences in species and type of embryos.

We also tried to knock down *IL17D* expression by RNA interference to examine its effects on cloned porcine embryo development (data not shown). However, only one siRNA with high specificity for targeting porcine *IL17D* mRNA was predicted by professional siRNA designing software due to the high homology of the *IL17D* gene to a gene called EEF1A lysine methyltransferase 1 in the pig genome. This potential *IL17D*-targeting siRNA could not decrease the porcine *IL17D* transcript level in transfected porcine cells and injected porcine SCNT embryos. Therefore, in the future, other strategies, such as *IL17D* gene knockout, should be employed to investigate the effects of inhibiting *IL17D* expression on porcine embryo development. In addition, future studies should test whether *IL17D* also participates in regulating the development of the SCNT embryos of other species, and whether IL17D can also improve the in vivo full-term developmental competence of cloned embryos.

## 5. Conclusions

In summary, IL17D improved the developmental competence of cloned porcine embryos by suppressing apoptosis and promoting EGA, which probably was related to the up-regulation of *GADD45B* expression. This study not only elucidated the functions of IL17D in early development of porcine SCNT embryos but also provided a new way to increase the developmental efficiency of cloned porcine embryos.

## Figures and Tables

**Table 1:** Experimental design of IL17D treatment of embryos.

	Types of Treated Embryos	Time Point of IL17D Addition	Groups	Evaluation Indexes
Experiment 1	SCNT	1-cell stage	NC, 5, 25, 50 and 100 ng/mL	Cleavage rate, blastocyst rate
Experiment 2	IVF	1-cell stage	NC, 50 ng/mL	Cleavage rate, blastocyst rate
Experiment 3	SCNT	4-cell stage	NC, 50 ng/mL	Cleavage rate, blastocyst rate
Experiment 4	SCNT	1-cell stage	NC, 50 ng/mL	Total cell number, number of apoptotic cells, percentage of apoptosis cells, apoptosis-related gene expression levels at blastocyst stage
Experiment 4	IVF	1-cell stage	NC, 50 ng/mL	Total cell number at blastocyst stage
Experiment 5	SCNT	1-cell stage	NC, 50 ng/mL	Transcriptome sequencing and qPCR validation at 4-cell stage

**Table 2:** Information of primers used for qRT-PCR.

Gene	Primer Sequences (5′-3′)	Product Size (bp)	Gene Accession Number
BCL2	F: TCGCCCTGTGGATGACTGAGTAC	131	XM_021099593
	R: AGACAGCCAGGAGAAATCAAATAGAGG		
BCL2L1	F: TGAGCAGGTATTGAACGAACTCTTCC	143	NM_214285
	R: CCATCCAAGTTGCGATCCGACTC		
TP53	F: GCCCATCCTCACCATCATCACAC	81	NM_213824
	R: GCACAAACACGCACCTCAAAGC		
CYCS	F: CGAGTGGTGGCTTGTCTGTTGAG	101	NM_001129970
	R: GGCACTGGGCACACTTCTGAAC		
BAX	F: ATCGGCTGCTGGGCTGGATC	124	XM_003127290
	R: ATGGTGAGCGAGGCGGTGAG		
BCL2L11	F: TGCCAAGCCTTCAACCATTATCTCAG	117	NM_001160074
	R: GTCTCCAATACGCCGTAACTCCTG		
BAD	F: CCGAGGAGGATGAAGGGACTGAG	138	XM_021082883
	R: AGGAACCCTGGAACTCGTCACTC		
FOS	F: GGAGTCGTGAAGACCATGCC	189	NM_001123113
	R: TAGCTGGTCTGTCTCCGCTTG		
JUN	F: AGGCGGAGAGGAAGCGTATGAG	145	NM_213880
	R: CTGAGCATGTTGGCGGTGGAC		
MAP3K8	F: CCAGTGAAGAGCCAGCAGTGTAC	150	XM_021064738
	R: GCAAGTCCTCCACGGTTCCATATC		
MYC	F: TCAACGTCAGCTTCACCAACAGG	85	NM_001005154
	R: AAGTTCTCCTCCTCGTCGCAGTAG		
GADD45B	F: TGAATTTGCTCTGTACCCAGGAACTC	108	XM_005654701
	R: GTAAGCCTCCCATCTCTCTTTCAGTG		
GAPDH	F: TGACCCCTTCATTGACCTCC	88	XM_021091114
	R: CTCCGCCTTGACTGTGCC		

**Table 3:** Developmental efficiency of SCNT embryos treated with IL17D at 1-cell stage for 48 h.

Groups	No. of Repetition	No. of Cultured SCNT Embryos	No. of Cleaved Embryos (%)	No. of Blastocysts (%)
NC	4	160	127 (79.12 ± 5.21 ^a^)	25 (15.48 ± 4.9 ^a^)
5 ng/mL IL17D	3	105	78 (74.29 ± 2.86 ^a^)	18 (17.14 ± 5.71 ^ab^)
25 ng/mL IL17D	3	107	76 (72.38 ± 7.19 ^a^)	21 (19.6 ± 2.54 ^ab^)
50 ng/mL IL17D	4	166	131 (81.62 ± 6.99 ^a^)	46 (27.59 ± 3.09 ^c^)
100 ng/mL IL17D	4	162	129 (79.47 ± 5.51 ^a^)	36 (22.06 ± 2.39 ^bc^)

NC, negative control. Values in the same column labeled with a same superscript have no significant difference with each other, labeled with a different superscript differ at *p* < 0.05.

**Table 4:** Developmental efficiency of IVF embryos treated with IL17D at 1-cell stage for 48 h.

Groups	No. of Repetition	No. of Cultured SCNT Embryos	No. of Cleaved Embryos (%)	No. of Blastocysts (%)
NC	6	298	211 (69.64 ± 8.76 ^a^)	45 (14.97 ± 4.14 ^a^)
50 ng/mL IL17D	6	289	210 (71.66 ± 8.32 ^a^)	67 (23.54 ± 3.34 ^b^)

NC, negative control. Values in the same column labeled with a same superscript have no significant difference with each other, labeled with a different superscript differ at *p* < 0.05.

**Table 5:** Developmental efficiency of SCNT embryos treated with IL17D at 4-cell stage for 48 h.

Groups	No. of Repetition	No. of Cultured SCNT Embryos	No. of Cleaved Embryos (%)	No. of Blastocysts (%)
NC	3	122	91 (74.57 ± 1.8 ^a^)	15 (12.28 ± 2.32 ^a^)
50 ng/mL IL17D	3	117	88 (75.21 ± 2.96 ^a^)	14 (11.97 ± 1.48 ^a^)

NC, negative control. Values in the same column labeled with a same superscript means they are not statistically different.

**Table 6:** Developmental efficiency of SCNT embryos injected with *GADD45B* expression plasmid.

Groups	No. of Repetition	No. of Cultured SCNT Embryos	No. of Cleaved Embryos (%)	No. of Blastocysts (%)
NC	3	113	88 (77.9 ± 5.94 ^a^)	5 (4.41 ± 1.48 ^a^)
10 ng/µL	3	113	77 (68.14 ± 0.49 ^a^)	8 (7.09 ± 1.58 ^a^)
50 ng/µL	3	111	78 (69.61 ± 4.45 ^a^)	10 (8.89 ± 5.49 ^a^)
100 ng/µL	3	112	82 (73.87 ± 7.8 ^a^)	12 (10.81 ± 2.7 ^b^)

Values in the same column labeled with a same superscript have no significant difference with each other, labeled with a different superscript differ at *p* < 0.05.

## Data Availability

The original data generated for this study are included in the article, further inquiries can be directed to the corresponding author.

## Supplementary Materials

The following are available online at www.mdpi.com/article/10.3390/ani11113062/s1, Figure S1: Validation of amplification efficiency of each pair of primers, Table S1: Primer-specific amplification efficiencies.

## Abbreviations

BAD, BCL2 associated agonist of cell death; BAX, BCL2 associated X; BCL2, B cell leukemia/lymphoma 2; BCL2L11, BCL2 like 11; BSA, bovine serum albumin; cDNA, complementary DNA; CDS, coding sequences; COCs, cumulus–oocyte complexes; CSF2, colony stimulating factor 2; DEGs, differentially expressed genes; DMEM, Dulbecco’s modified eagle medium; DPBS, Dulbecco’s phosphate-buffered saline; EGA, embryonic genome activation; EGFP, enhanced green fluorescent protein; FBS, fetal bovine serum; FOS, Fos proto-oncogene, AP-1 transcription factor subunit; FPKMs, fragments per kilobase of exon model per million mapped reads; GADD45B, growth arrest and DNA damage inducible beta; IGF1, insulin like growth factor 1; IL17D, interleukin 17D; IVF, in vitro fertilization; JUN, Jun proto-oncogene, AP-1 transcription factor subunit; MAPK, mitogen-activated protein kinase; MAP3K8, mitogen-activated protein kinase kinase kinase 8; mTBM, modified Tris-buffered medium; MYC, MYC proto-oncogene, bHLH transcription factor; PZM-3, porcine zygote medium 3; qRT-PCR, Quantitative real-time polymerase chain reaction; TUNEL, terminal deoxynucleotidyl transferase (TdT)-mediated 2′-deoxyuridine-5′-triphosphate-digoxigenin nick-end labeling; SCNT, somatic cell nuclear transfer.
