# Supplementary material for: Interleukin 17D Enhances the Developmental Competence of Cloned Pig Embryos by Inhibiting Apoptosis and Promoting Embryonic Genome Activation
Source: Animals (Basel). 2021 Oct 26;11(11):3062. doi: 10.3390/ani11113062 (PMC8614321; doi:10.3390/ani11113062)
Supplement: Supplementary file 1 [file animals-11-03062-s001.zip › animals-1327811-supplementary(1).pdf]

Table S1. Primer-specific amplification efficiencies.

Gene symbol	Slope	R^2	Amplification efficiency
BCL2	-3.2213	0.9993	104.38%
BCL2L1	-3.3389	0.9992	99.30%
TP53	-3.2757	0.9979	101.97%
CYCS	-3.1848	0.9985	106.06%
BAX	-3.2486	0.9992	103.15%
BCL2L11	-3.3216	0.9986	100.01%
BAD	-3.3388	0.9973	99.30%
FOS	-3.0135	0.9881	114.70%
JUN	-3.3566	0.9983	98.57%
MAP3K8	-3.3415	0.9973	99.19%
MYC	-3.4039	0.9952	96.69%
GADD45B	-3.4236	0.997	95.93%
GAPDH	-3.4413	0.9999	95.25%

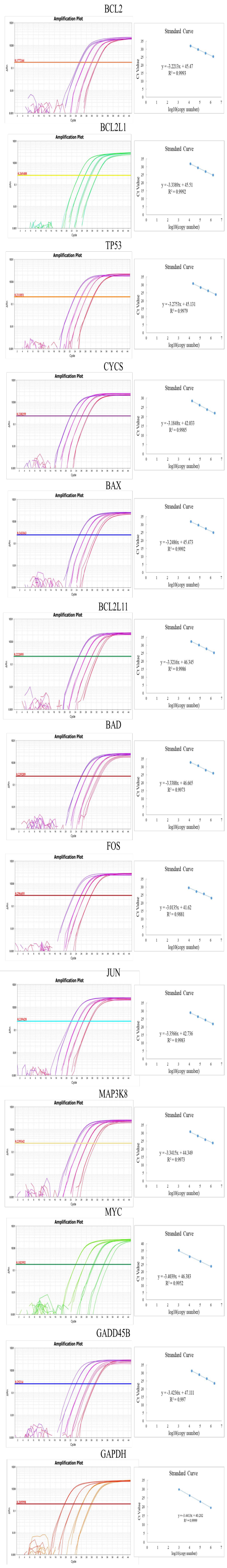
BCL2
Amplification Plot
Strandard Curve
y = -3.2213x + 45.47
R² = 0.9993
log10(copy number)
Ct Value
BCL2L1
Amplification Plot
Strandard Curve
y = -3.3389x + 45.51
R² = 0.9992
TP53
Amplification Plot
Strandard Curve
y = -3.2753x + 45.131
R² = 0.9979
CYCS
Amplification Plot
Strandard Curve
y = -3.1848x + 42.033
R² = 0.9985
BAX
Amplification Plot
Strandard Curve
y = -3.2486x + 45.473
R² = 0.9992
BCL2L11
Amplification Plot
Strandard Curve
y = -3.3216x + 46.345
R² = 0.9986
BAD
Amplification Plot
Strandard Curve
y = -3.3388x + 46.665
R² = 0.9973
FOS
Amplification Plot
Strandard Curve
y = -3.0135x + 41.62
R² = 0.9881
JUN
Amplification Plot
Strandard Curve
y = -3.3566x + 42.736
R² = 0.9983
MAP3K8
Amplification Plot
Strandard Curve
y = -3.3415x + 44.349
R² = 0.9973
MYC
Amplification Plot
Strandard Curve
y = -3.4039x + 46.383
R² = 0.9952
GADD45B
Amplification Plot
Strandard Curve
y = -3.4236x + 47.111
R² = 0.997
GAPDH
Amplification Plot
Strandard Curve
y = -3.4413x + 40.202
R² = 0.9999

Figure S1. Validation of amplification efficiency of each pair of primers.
